# DNA damage response of haematopoietic stem and progenitor cells to high-LET neutron irradiation

**DOI:** 10.1038/s41598-021-00229-2

**Published:** 2021-10-21

**Authors:** Monique Engelbrecht, Roya Ndimba, Maryna de Kock, Xanthene Miles, Shankari Nair, Randall Fisher, Peter du Plessis, Julie Bolcaen, Matthys Hendrik Botha, Elbie Zwanepoel, Simon Sioen, Ans Baeyens, Jaime Nieto-Camero, Evan de Kock, Charlot Vandevoorde

**Affiliations:** 1 Department of Medical Bioscience, University of the Western Cape, Faculty of Science, Robert Sobukwe Road, Bellville, Cape Town, 7535 South Africa; 2 Nuclear Medicine Department, Radiation Biophysics Division, NRF-iThemba LABS, Old Faure Road, Faure, Cape Town, 7131 South Africa; 3 Department of Obstetrics and Gynaecology, Stellenbosch University, Faculty of Medicine and Health Sciences, Clinical Building, Francie van Zijl Drive, Parow Valley, Cape Town, 7500 South Africa; 4 Department of Obstetrics and Gynaecology, Karl Bremer Hospital, Frans Conradie Drive, Bellville, 7530 South Africa; 5 Radiobiology, Department of Human Structure and Repair, Ghent University, Corneel Heymanslaan 10, 9000 Gent, Belgium

**Keywords:** Biological techniques, Biophysics, Cancer, Cell biology

## Abstract

The radiosensitivity of haematopoietic stem and progenitor cells (HSPCs) to neutron radiation remains largely underexplored, notwithstanding their potential role as target cells for radiation-induced leukemogenesis. New insights are required for radiation protection purposes, particularly for aviation, space missions, nuclear accidents and even particle therapy. In this study, HSPCs (CD34^+^CD38^+^ cells) were isolated from umbilical cord blood and irradiated with ^60^Co γ-rays (photons) and high energy p(66)/Be(40) neutrons. At 2 h post-irradiation, a significantly higher number of 1.28 ± 0.12 γ-H2AX foci/cell was observed after 0.5 Gy neutrons compared to 0.84 ± 0.14 foci/cell for photons, but this decreased to similar levels for both radiation qualities after 18 h. However, a significant difference in late apoptosis was observed with Annexin-V^+^/PI^+^ assay between photon and neutron irradiation at 18 h, 43.17 ± 6.10% versus 55.55 ± 4.87%, respectively. A significant increase in MN frequency was observed after both 0.5 and 1 Gy neutron irradiation compared to photons illustrating higher levels of neutron-induced cytogenetic damage, while there was no difference in the nuclear division index between both radiation qualities. The results point towards a higher induction of DNA damage after neutron irradiation in HSPCs followed by error-prone DNA repair, which contributes to genomic instability and a higher risk of leukemogenesis.

## Introduction

Radiosensitivity refers to the relative susceptibility of cells, tissues, organs and organisms to the harmful effects of ionising radiation (IR)^1^. In humans, one of the most radiosensitive tissues is the haematopoietic system^2^. Haematopoiesis is the process of blood cell formation, originating from a common precursor, the haematopoietic stem and progenitor cell (HSPC), which reside in a tightly controlled bone marrow (BM) niche that regulates the quiescence, proliferation and differentiation of HSPCs^3,4^. According to the UNSCEAR 2013 report, children are generally identified to carry a higher risk for radiation-induced malignancies in comparison to adults. This risk is quantified to be 2–3 times higher for specific solid tumours and 3–5 times higher for haematological malignancies, including leukaemia^5^. A causative link between IR exposure and leukaemia risk has been extensively studied amongst the Japanese Atomic bomb survivors^6,7^. This provided compelling evidence that high doses of IR lead to significant increases in the incidence of several types of leukaemia, including acute lymphocytic leukaemia (ALL), acute myeloid leukaemia (AML) and chronic myeloid leukaemia (CML) subtypes; but not for chronic lymphocytic leukaemia (CLL)^8^. For children exposed during the Nagasaki and Hiroshima bombings, the rates of leukaemia incidence were particularly high, especially for ALL, which is the most common type of paediatric cancer^7,9^. In addition to these epidemiological studies on the mainly high and acute IR exposures during the atomic bombs, several studies have also linked low-dose IR exposures to increased leukaemia risks. Examples are the elevated risk to develop leukaemia after diagnostic CT scans during childhood, as well as the large international nuclear worker study (INWORKS), where repeated and protracted low-dose IR exposures were associated with an elevated leukaemia risk^10–15^. The target cells for radiation-induced leukaemia are most likely the HSPCs, since their long-life span allows the accumulation of radiation damage which could compromise their genomic integrity and potentially give rise to leukemogenesis^16–18^. Several experimental studies on HSPCs have already illustrated that low dose IR exposure can lead to impaired self-renewal capacity, long-term deleterious effects and cell death^16,19,20^. Despite the growing number of studies on the radiosensitivity of HSPCs, definitive conclusions regarding radiation-induced cell death, DNA repair, and genomic stability in these rare quiescent cells are lacking^21–25^. In particular, experiments on the DNA damage response of HSPCs to high linear energy transfer (LET) radiation, such as neutrons and carbon ions, remain scarce^26–29^.

The leukaemia risks associated with exposure to neutron radiation are also of increasing interest with regards to aviation, future space missions, nuclear accidents and even modern particle therapy. In all these scenarios, being it occupational, accidental or medical exposures; neutrons can undergo a wealth of nuclear reactions, giving rise to a complex mixed field of secondary charged particles which can induce significant biological damage. The associated health risks were addressed by Baiocco et al. in the EU FP7 project ANDANTE, where a track structure model was developed to investigate the patterns of damage at cellular levels^30–32^. For radiation protection purposes, radiation weighting factors (w_R_) are generally used to convert the physical absorbed dose (Gy) into an equivalent dose (Sv), in order to estimate radiation-induced cancer risks^33^. For neutrons, the w_R_ is based on old experimental relative biological effectiveness (RBE) data on the induction of dicentric chromosomes in human lymphocytes by neutron radiation with energies < 20 MeV^34^. As a consequence of the fact that the secondary particle field induced by neutrons varies with neutron energy, the neutron RBE will also depend on the energy. Therefore, considerable uncertainty remains on how the neutron RBE varies at higher neutron energies (> 20 MeV), but also with dose and dose rate^35^. This has resulted in successive corrections and changes in radiation protection standards and neutron w_R_ over time^31^. It is generally accepted that the maximum biological effect can be observed for 1 MeV neutrons^36^. However, RBE values derived from human data and exposure to high-energy neutrons remain scarce and are urgently warranted^32,37–41^.

The growing interest in high-energy neutron radiobiology is mainly driven by the increase in proton therapy (PT) facilities around the world and our desire to explore space beyond lower Earth orbit with manned mission to the Moon and Mars. Despite the dose sparing properties of PT, secondary neutrons are inevitably produced outside the primary field^42–46^. While it is anticipated that the absorbed dose resulting from these secondary neutrons is small, especially for more recent pencil-beam scanning PT facilities, the uncertainty around the high neutron RBE remains a topic of concern, particularly for paediatric patients^47^. During space missions, secondary neutrons are produced as a result of nuclear interactions with the spacecraft wall and with the human body^48^. Radiation-induced leukaemia represents about 15% of the total cancer risk from space radiation for the upcoming interplanetary space missions^49^. Furthermore, the exposure of aircrew to cosmic radiation has been recognised as an occupational health risk and remains a topic of active debate for legal radiation protection regulations^50^. The absorbed radiation dose of the aircrew is considered to be low, but typically 30–50% is coming from high-LET radiation, such as neutrons^50^. For male cockpit crew members with a long flying history (> 5000 h) a significantly increased frequency of AML has been observed^51^.

The clear association between radiation exposure, particularly during childhood but also later in life, and the subsequent risk of developing leukaemia, has resulted in a growing number of studies on the radiosensitivity of HSPCs. However, to the best of our knowledge, this is the first in vitro study to investigate the response of HSPCs to higher-energy neutron irradiation. Human HSPCs can be identified by means of the CD34 surface glycoprotein marker, leading to their designation as CD34^+^ cells^52,53^. In this study, CD34^+^ cells were isolated from umbilical cord blood (UCB) and their DNA damage response to high-LET p(66)/Be(40) neutrons and low-LET Cobalt-60 (^60^Co) gamma (γ)-rays was investigated.

## Results

### Radiation-induced chromosomal damage in CD34^+^ cells

**Figure 1:**
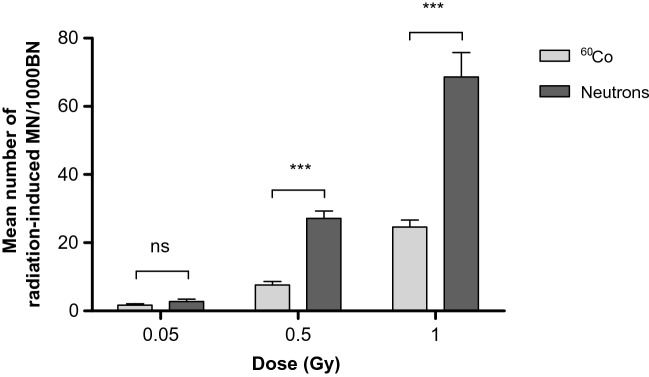
Mean number of micronuclei (MN) in CD34^+^ cells (n = 12) induced by different doses (0.05, 0.5 and 1 Gy) of ^60^Co γ-rays and p(66)/Be(40) neuron irradiation. The number of MN induced by the irradiation was obtained by subtracting the mean number of MN in the non-irradiated controls (0 Gy) from the mean MN number scored in the irradiated samples. MN yields were significantly higher post-neutron irradiation compared to ^60^Co γ-rays (***p < 0.001) for the 0.5 Gy and 1 Gy dose (***p < 0.001) but not for the 0.05 Gy dose (ns). Error bars represent the standard error of the mean (SEM) of the 12 different donors for each radiation quality. At least 1000 BN cells were scored for each donor per condition. No significant difference is indicated by ns.

**Figure 2:**
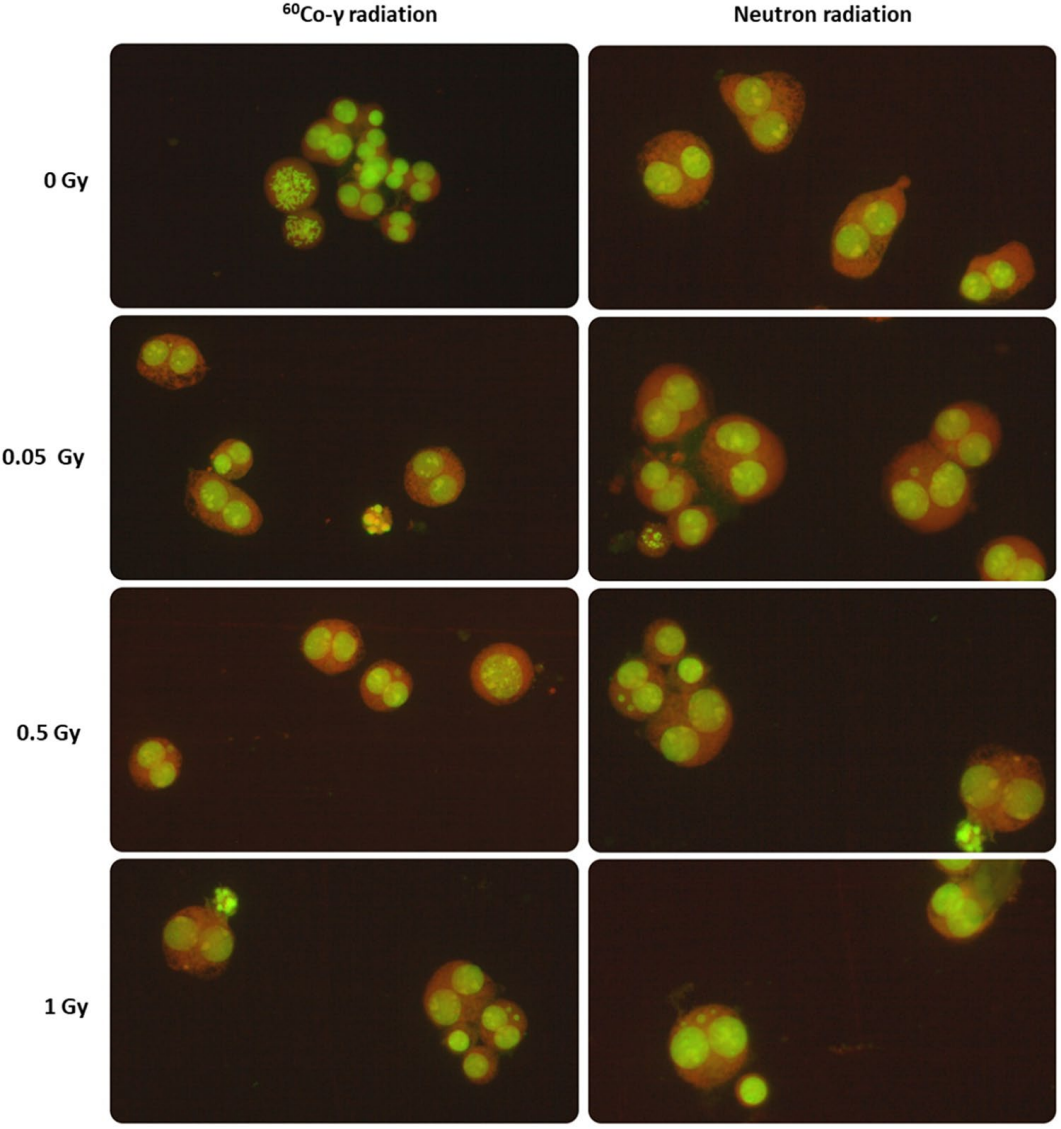
Characteristic appearance of human CD34^+^ cells for the different radiation doses that were investigated as part of the CBMN assay. The images illustrate the BN CD34^+^ cells containing micronuclei after low-LET ^60^Co γ-rays (left panel) and high-LET neutrons (right panel) exposure. The images were captured from a fluorescent Zeiss Axio Imager A1 microscope, at 20× magnification.

The cytokinesis-block micronucleus (CBMN) assay has become a well-established standard method for measuring DNA damage in human peripheral blood lymphocytes (PBL) after IR exposure^54^. Micronuclei (MN) are extra-nuclear bodies that contain damaged chromosome fragments and/or whole chromosomes that were not incorporated into the nucleus after cell division^55^. For this study, a previously established micro-culture CBMN assay was adapted in order to expose isolated CD34^+^ cells of the same donors (n = 12) to ^60^Co γ-rays and p(66)/Be(40) neutron irradiation after cryopreservation^23^. Figure 1 shows the number of radiation-induced MN, reflecting chromosome breakage or whole chromosome loss after exposure to radiation doses of 0.05, 0.5 and 1 Gy. Although there was no statistically significant difference in MN yields at the lowest dose of 0.05 Gy between ^60^Co γ-rays and neutrons (*p* > 0.05); a significant higher MN frequency was observed at 0.5 and 1 Gy for neutrons (*p* < 0.001). The lowest dose of 0.05 Gy yielded an average MN frequency of 2.79 ± 0.38 MN/1000 BN cells and 3.92 ± 0.74 MN/1000 BN cells after ^60^Co γ-rays and neutron irradiation respectively, which was significantly higher than the low average control values (0 Gy) of 1.25 ± 0.33 MN/1000 BN cells for ^60^Co γ-rays (*p* < 0.01) and 1.46 ± 0.23 MN/1000 BN cells for neutron irradiation experiments (*p* < 0.01). Not all the irradiation experiments could be performed on the same day, therefore separate control (0 Gy) cultures were set-up for both radiation qualities. Figure 2 shows the characteristic appearance of human CD34^+^ cells for the different radiation doses as part of the CBMN assay. The RBE is normally calculated at the same level of biological effect, but the number of dose points in this study was too limited to fit a dose response curve. Therefore, a biological enhancement ratio was calculated and presented in Table 1. This is the ratio of the average radiation-induced MN frequency for neutron radiation over ^60^Co γ-rays, ranging between 1.61 and 3.55 for the radiation doses used in these experiments, with a maximum observed at 0.5 Gy (Table 1). No statistical significant difference was observed in biological enhancement ratio at the different radiation doses, except for 0.05 Gy versus 0.5 Gy (*p* = 0.0134).

**Table 1:** Ratio of the mean number of radiation-induced MN (n = 12) at different dose points (Neutrons/^60^Co).

Dose (Gy)	0.05	0.5	1
Biological enhancement ratio	1.61 ± 1.26	3.55 ± 0.53	2.79 ± 0.47

The propagation of uncertainty was calculated based on the standard deviations on the induced average MN frequencies.

**Figure 3:**
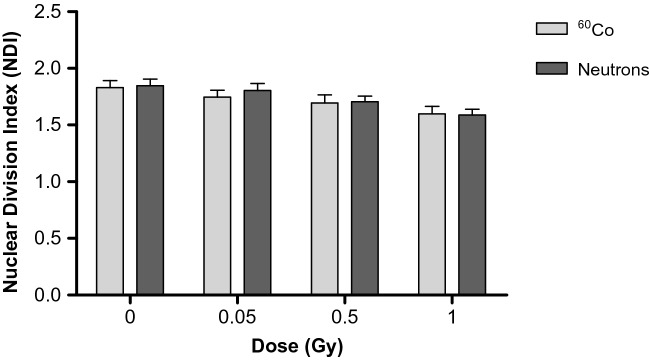
The nuclear division index (NDI) was calculated to compare the proliferation status of the micro-culture CBMN assay for the CD34^+^ samples irradiated with different radiation qualities with doses of 0, 0.05, 0.5 and 1 Gy. Error bars represent the standard error of the mean (SEM) of the 12 different donors for each radiation quality. At least 500 viable cells were manually scored for each donor per condition.

In order to assess the impact of the two radiation qualities and the different radiation doses on the CD34^+^ cell proliferation, the nuclear division index (NDI) was calculated. This value reflects the mitotic activity of the CD34^+^ cells by quantifying the proportion of mitotic, viable cells and it gives a general indication of a cytotoxic effect of the irradiation exposure on the CD34^+^ cell proliferation. Cells with extensive chromosomal damage might fail to undergo cell division and would not be reflected in the final number of BN cells that are scored. Although there is an apparent decreasing trend in the NDI with increasing dose for both radiation qualities, no statistically significant difference was found between ^60^Co γ-ray and neutron irradiation (*p* > 0.05) (Fig. 3). In addition, all average NDI values were between 1.0 and 2.0, illustrating that the CD34^+^ CBMN culture method was successful^54^. There was a significance decrease in the NDI of each individual donor when the absorbed dose of 1 Gy was compared to the control (0 Gy) NDI for each radiation quality (*p* < 0.001). Overall, the average MN frequency increased with the radiation dose and was significantly higher after neutron irradiation compared to ^60^Co γ-rays, while the average NDI remained consistent for both radiation qualities.

### DNA double-strand breaks (DSBs) formation and repair after ^60^Co-γ-rays versus neutron irradiation

**Figure 4:**
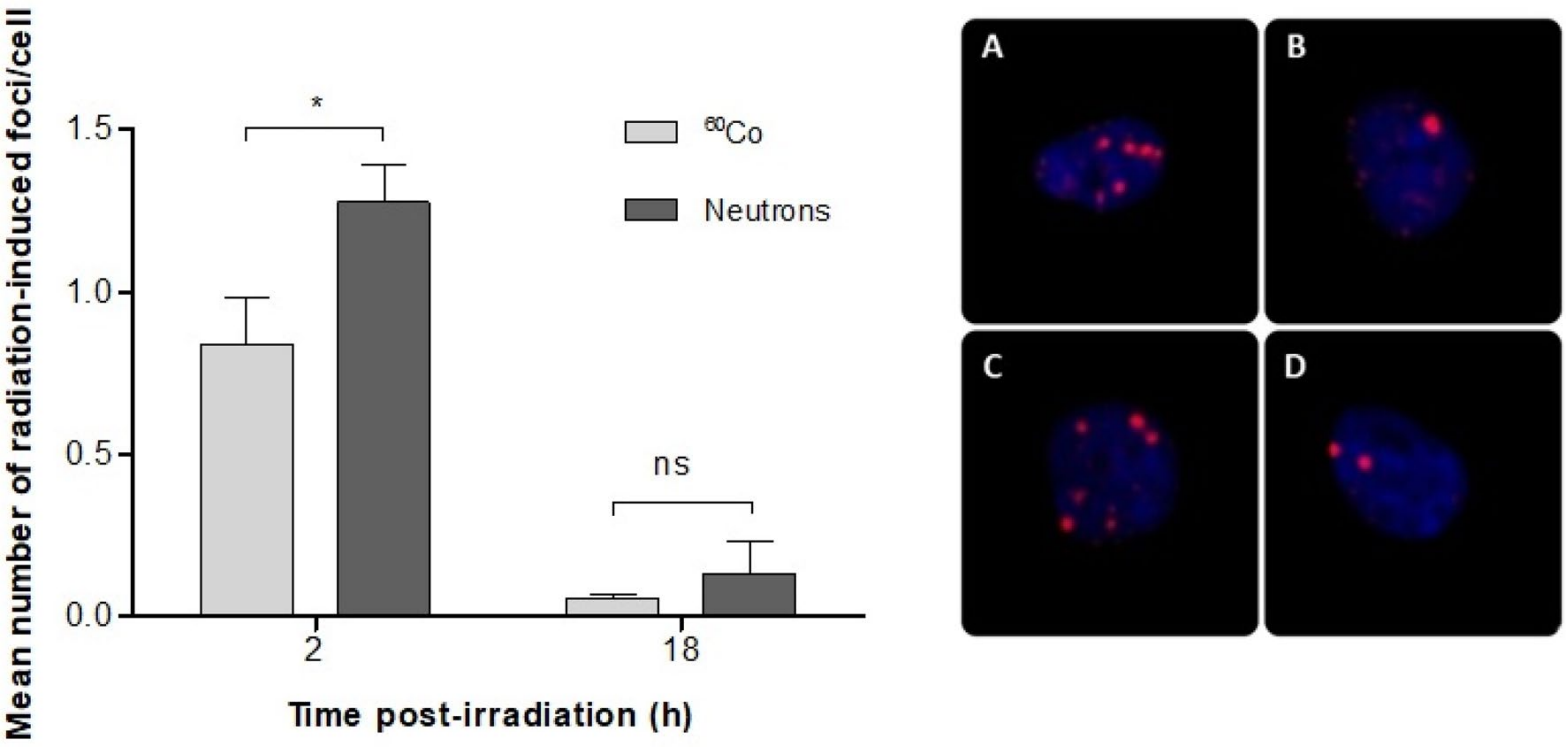
Mean number of radiation-induced γ-H2AX foci per CD34^+^ cell at 2 and 18 h post-irradiation with 0.5 Gy. The number of radiation-induced γ-H2AX foci was obtained by subtracting the mean number of γ-H2AX foci in the non-irradiated controls from the mean γ-H2AX foci number scored in the irradiated samples. The number of radiation-induced γ-H2AX foci was significantly different between ^60^Co γ-rays (n = 6) and neutron (n = 9) radiation at 2 h (*p < 0.05), while after 18 h no significant difference (*p* > 0.05) was observed between the two radiation qualities. Error bars represent standard error of the mean (SEM).The images (**A**–**D**) on the right-side show immunofluorescence staining of γ-H2AX foci with a TRITC-conjugated secondary antibody (red). The foci represent the residual DNA DSBs at 2 h (**A** and **C**) and 18 h (**B** and **D**) after 0.5 Gy ^60^Co γ-rays (**A** and **B**) and neutron irradiation (**C** and **D**).

The γ-H2AX foci assay is considered to be a highly sensitive technique to evaluate DNA DSB formation and repair following exposure to IR. As shown in Fig. 4, the initial γ-H2AX foci formation 2 h after exposure to neutrons was significantly higher than after ^60^Co γ-ray irradiation (*p* = 0.049). This indicates that high-LET neutron irradiation induced a higher number of initial DNA DSBs in CD34^+^ cells compared to ^60^Co γ-rays. However, while it is expected that the repair kinetics of the more complex DNA damage induced by neutron irradiation would be slower compared to DNA DSB repair observed for ^60^Co γ-rays, no statistically significant difference could be observed at 18 h post-irradiation between both radiation qualities. A residual number of 0.353 ± 0.149 γ-H2AX foci/cell and 0.542 ± 0.106 γ-H2AX foci were observed at 18 h post-irradiation for ^60^Co γ-rays and neutrons, respectively, which was elevated but not significantly different from the control values of 0.253 ± 0.077 foci/cell for ^60^Co γ-rays (*p* = 0.937) and 0.411 ± 0.085 foci/cell for neutrons (*p* = 0.436).

### Radiation-induced apoptosis in CD34^+^ cells

**Figure 5:**
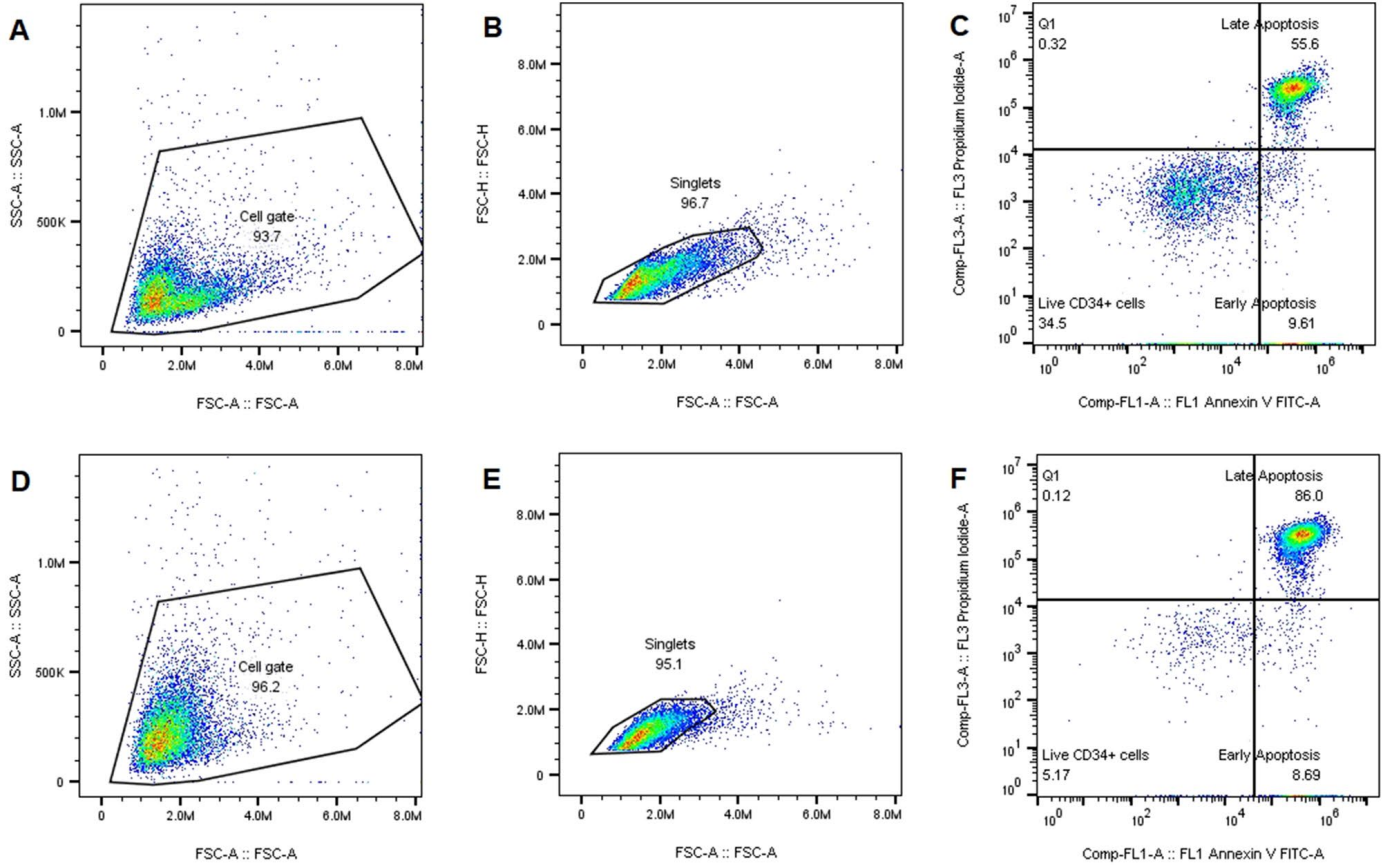
The gating strategy for the Annexin-V/PI apoptosis analysis. CD34^+^ cells were gated on forward (FSC) versus side scatter (SSC) to select the cell population (**A** and **D**). Next, the cells were gated on FSC-Height (FSC-H) vs FSC-Area (FSC-A) to exclude all the doublets and to generate the singlets gate (**B** and **E**). Finally, all the subpopulations were analysed on the Annexin V-FITC versus PI scatter for live, early and late apoptosis at 18 h post-irradiation (**C** and **F**). The upper part (**A**–**C**) represents the gating strategy of CD34^+^ cells irradiated with a low dose of neutrons at 0.5 Gy; and the lower part (**D**–**F**) CD34^+^ cells irradiated with a high dose at 3 Gy which resulted in a higher percentage of late apoptosis.

When the amount or severity of radiation-induced DNA damage in the CD34^+^ cells surpasses the repair capacity, the cells can undergo programmed cell death, such as apoptosis. In this study, the Annexin-V/PI assay was used to assess the fraction of live (Annexin-V^−^/PI^−^), early (Annexin-V^+^/PI^−^) and late (Annexin-V^+^/PI^+^) apoptotic cells at 18 and 42 h post-irradiation. The flow cytometry gating strategy is presented in Fig. 5. As depicted in Table 2, there is a distinct decrease in the percentage of living CD34^+^ cells with time post-irradiation. While there was no significant difference between the percentage of living cells for both radiation qualities (*p* > 0.05), neutron radiation induced a larger decrease in living cells compared to ^60^Co γ-rays. At 18 h post-radiation, the highest dose of 3 Gy resulted in 46.71 ± 7.25% and 31.26 ± 4.87% living cells after ^60^Co γ-rays and neutron irradiation, respectively. While at 42 h post-irradiation, the fraction of living cells decreased to 26.85 ± 5.33% and 19.12 ± 4.28% after 3 Gy of ^60^Co γ-rays and neutron irradiation, respectively. However, no significant difference in early apoptosis was observed between ^60^Co γ-rays compared to neutrons at any dose. In the analysis of late apoptosis, see Table 2, there was a significant increase from 18 to 42 h post-irradiation for both radiation qualities (*p* < 0.01). Furthermore, a statistically significant difference in late apoptosis was observed between ^60^Co γ-rays and neutron irradiations (*p* < 0.05). Overall, the late apoptosis levels after exposure to high-LET neutrons was higher in comparison to low-LET ^60^Co γ-rays (Table 2). For example, 18 h post-irradiation, the percentage of late apoptosis at a dose of 3 Gy was 43.17 ± 6.14% for ^60^Co γ-rays and 55.55 ± 4.87% for neutron irradiation.

**Table 2:** This table represents mean percentage (standard error of the mean (SEM)) of living, early and late apoptotic CD34^+^ cells of the same donors were irradiated with three different radiation doses of 0, 0.5, 1 and 3 Gy of ^60^Co γ-rays and neutrons after 18 and 42 h (n = 13).

Dose (Gy)	Hours (h)	^60^Co γ-rays			Neutrons		
		Live cells	Early apoptosis	Late apoptosis	Live cells	Early apoptosis	Late apoptosis
0	18	61.03 ± 5.77	14.62 ± 2.41	26.24 ± 4.20	57.18 ± 6.04	15.46 ± 2.66	26.92 ± 3.99
0.5		45.25 ± 7.23	14.23 ± 2.03	36.21 ± 7.60	44.58 ± 6.88	14.88 ± 1.54	40.73 ± 5.88
1		46.06 ± 9.00	11.88 ± 2.20	38.94 ± 9.10	38.64 ± 7.05	14.975 ± 1.75	45.97 ± 6.22
3		46.71 ± 7.25	11.43 ± 1.37	43.17 ± 6.10	31.26 ± 4.87	10.50 ± 0.95	55.55 ± 4.87
0	42	48.78 ± 6.40	29.60 ± 8.01	26.38 ± 4.50	50.08 ± 6.15	28.28 ± 4.10	29.50 ± 3.92
0.5		27.19 ± 4.92	26.85 ± 11.15	44.62 ± 6.20	25.86 ± 4.21	28.40 ± 10.69	52.20 ± 3.92
1		24.39 ± 4.88	23.90 ± 10.36	53.55 ± 6.20	20.83 ± 4.25	26.27 ± 12.30	59.32 ± 5.03
3		26.85 ± 5.33	22.18 ± 10.74	52.35 ± 7.00	19.12 ± 12.00	12.00 ± 3.05	61.18 ± 5.67

## Discussion

The clear differences in excess leukaemia risk between the Hiroshima and Nagasaki atomic bomb survivors, supports the general finding that neutrons are more effective than γ-rays in causing radiation-induced leukaemia^7,56^. Since HSPCs are characterised by a long life-span which imparts a propensity to accumulate mutations and other alterations that could trigger leukemogenesis, they became the cell type of interest to study the underlying mechanisms of radiation-induced leukaemogenesis^17,57,58^. However, the number of studies which investigated the response of HSPCs to high-LET and high-energy neutrons remain limited^26–29,59^. Therefore, the aim of the present study was to address this knowledge gap by investigating how HSPCs respond to high-energy neutron radiation in comparison to a frequently studied reference radiation quality, ^60^Co γ-rays.

It is well known that high-LET neutron radiation is more effective than sparsely ionising low-LET radiation to induce cytogenetic damage^60^, which was also confirmed in the present study (Fig. 1). Similar to previous studies, the number of radiation-induced MN strongly correlates with radiation dose and depends on the radiation quality^61^. Becker et al*.* reported a higher frequency of chromosomal aberrations in human CD34^+^ cells after low-LET X-rays and high-LET carbon ions (29 keV/µm)^26^. In addition, the fraction of complex-type aberrations was higher following carbon ion exposure. A RBE value of 1.4 was observed at a biological effect of 1 aberration/cell^26^. In this study, the biological enhancement ratio ranged from 1.61 to 3.55 depending on the neutron radiation dose (Table 1). This result exceeds the relative biological effectiveness (RBE) values reported by Becker et al*.* and contradicts the general rule that the RBE increases at lower doses. However, this enhancement ratio is not a valid substitute for RBE and dose response curves are required to determine α and β parameters to calculate the RBE at the desired level of biological effect. Vandersickel et al. reported RBE values that ranged between 3.6 and 1.6 for peripheral blood lymphocytes (PBL) in the dose range of 0.05–2 Gy using the same p(66)/Be(40) neutron irradiation facility. Rall et al. used premature chromosome condensation to study the rejoining of radiation-induced chromatid breaks 9 h after 2 Gy irradiation with photons (250 kV X-rays) and heavy ions (nitrogen, carbon, titanium, and calcium) in the LET range of 45–180 keV/μm in PBL and CD34^+^ cells^29^. For X-ray irradiation, more than 50% of the chromatid breaks were repaired within 1–2 h post-irradiation. However, the rejoining of chromatid breaks was slower after 2 Gy of very high-LET irradiation (calcium and titanium ions, 180 and 150 keV/µm, respectively)^29^.

In order to confirm that neutron radiation did not affect the outcome and quality of the CBMN assay in this study, the average nuclear division index (NDI) values were determined for both irradiation qualities (Fig. 3). The NDI for PBL is presumed to be in the range of 1.3–2.2^54^. This is in line with the current results for isolated CD34^+^ cells, with a comparable NDI of 1.84 ± 0.06 for the unirradiated cultures, which is also in accordance with previous publications which reported values of 1.58 ± 0.13^23^ and 1.58 ± 0.10^62^. No significant difference could be observed in the mean NDI for the two different radiation qualities (*p* > 0.05), illustrating that the low radiation doses did not impact the proliferation capacity of the CD34^+^ cells and the CBMN assay provides a true reflection of the radiation-induced cytogenetic damage. It is important to note that the background MN frequency of the control CD34^+^ cultures was only 1.35 ± 0.20 MN/1000 BN cells in this study. This is much lower than the background MN value of 17 ± 10 MN/1000 BN cells that was reported for isolated PBL cultures of adult donors using the same manual scoring method^63^. The low background frequencies are however in agreement with previous studies which used CD34^+^ or PBLs isolated from UCB, which reflect the low exposure of UCB cells to genotoxic agents at this early stage of life^23,64,65^. Due to the low background values, it was possible to detect radiation-induced MN after a low dose of 0.05 Gy, bordering the sensitivity limit of the CBMN assay. However, large scale biomonitoring studies were also able to demonstrate genetic damage with the CBMN assay for accumulated occupational doses of 50 mGy^66^. A challenge in this particular study was to obtain a sufficient number of CD34^+^ cells from one donor to perform several CBMN cultures in parallel with different doses and radiation qualities. Different culturing methods have been used by research groups to solve this problem. One group decided to pool CD34^+^ cells of different donors, while others expand the CD34^+^ cells in culture. The latter was described by Hintzsche et al., who pre-cultured the CD34^+^ cells upon thawing for 4 days before experimental treatment in order to obtain feasible cell numbers^67^. This resulted in a 7- to 10-fold increase in cell number, but this method increases the chance of further differentiation. In the current study, a modified version of a previously developed protocol was used^23^, where approximately 100,000 of CD34^+^ cells were cultured in a small culture volume (500 μL) for 70 h.

A higher number of DNA DSBs was observed with the γ-H2AX foci assay in CD34^+^ cells after neutron irradiation compared to ^60^Co γ-rays at 2 h post-irradiation. However, at the later timepoint of 18 h similar levels of residual γ-H2AX foci were observed despite the much higher initial number of γ-H2AX foci for neutron radiation at 2 h (Fig. 4). This observation, together with the higher level of neutron-induced MN/1000 BN cells compared to the ^60^Co radiation in this study, suggests a fast repair in CD34^+^ cells after neutron irradiation with a higher error rate compared to low LET ^60^Co γ-ray irradiation. The γ-H2AX foci results at the early time point contradict the results of a previous study performed on isolated PBL, where a significantly higher number of γ-H2AX foci was observed 2 h post-irradiation with ^60^Co γ-rays compared to p(66)/Be(40) neutrons^68^. It illustrates potential underlying differences in DNA repair processes between CD34^+^ cells and isolated lymphocytes, which is confirmed by several other studies^23–25,69–72^. The selection of the 2 h time point for initial damage is rather unusual, since the maximum number of γ-H2AX foci is supposed to be formed at 30 min post-irradiation. However, this decision was based on a previous observation, where DNA DSB repair was studied in isolated lymphocytes using the same p(66)/Be(40) beam and the maximum number of γ-H2AX foci was only observed at 2 h post-irradiation^35^. The results at 18 h post-irradiation were not expected, since previous studies have indicated that approximately 20–40% of the DNA DSBs induced by low-LET radiation exposure are complex and clustered, while this increases to approximately ~ 70% for high-LET radiation^73,74^. Given that clustered DNA damage is often poorly or not repaired, the clustered DNA damage is most probably responsible for the greater mutagenic and cytotoxic effects of high-LET radiation^73^. Therefore, one would expect a higher fraction of residual γ-H2AX foci after 18 h for high-LET neutrons compared to low-LET ^60^Co γ-rays, which was not the case. However, the number of residual γ-H2AX foci in isolated lymphocytes was also not significantly different after 24 h in the study of Vandersickel et al. and the repair half-life was very similar for the two radiation qualities, namely 2.8 and 3 h for ^60^Co γ-rays and p(66)/Be(40) neutrons respectively^68^. The latter agrees with the observations made here at 18 h post-irradiation for CD34^+^ cells, where no significant difference was observed between the two radiation qualities. However, the study of Heylmann et al. illustrates that apoptosis and DNA DSB repair results obtained with PBL or lymphocyte subsets cannot be simply projected on CD34^+^ cells^72^. Different lymphocyte subsets and CD34^+^ cells were isoalted from peripheral blood and differences in repair kinetics were observed. The CD34^+^ cells showed a faster repair after 4 h in comparison with T-lymphocytes (CD3^+^). In both CD34^+^ progenitor cells and T-lymphocytes, the residual values at 24 h returned to normal in comparsion with 0 h. In the current study, the decrease of γ-H2AX foci over time indicates the DNA DSB repair compentence of CD34^+^ cells, but the residual values at 18 h were still significantly higher than the control (0 Gy) values. Rall et al*.* evaluated γ-H2AX foci formation in stimulated CD34^+^ cells at different time points post-irradiation with 2 Gy of X-rays and high-LET iron ions and did observe a significant difference in residual DNA damage at 24 h for both radiation qualities ^29^. However, in contrast to the current study, the CD34^+^ cells were stimulated and the LET of the iron ion beam is expected to be much higher than the neutron beam used in this study. To the best of our knowledge, only a limited number of studies investigated DNA DSB formation and repair in CD34^+^ cells after high-LET radiation, which limits the comparison of the γ-H2AX foci results mainly to experiments with low-LET radiation. The endogenous γ-H2AX foci levels were low in the isolated CD34^+^ cells, with an average value of 0.319 ± 0.052 γ-H2AX foci/cell, which is in line with previous studies for CD34^+^ cells isolated from UCB^23,75^. The γ-H2AX foci study of Vasilyev et al*.* who also evaluated residual damage at 18 h post-irradiation as well as other studies with low-LET radiation at 24 h suggest a fast DNA damage repair capacity in CD34^+^ cells^70^.

After IR exposure, an appropriate DNA damage response is the initial attempt of the cell to repair radiation-induced lesions, but if this damage is too extensive, a signalling cascade will trigger cell death to prevent genomic instability. For early apoptosis, the percentage of cells remained at similar levels with increasing dose and no significant difference could be overserved between both radiation qualities (Table 2). The levels of late apoptosis gradually increased with dose for both low- and high-LET radiation (Table 2), and a significant difference was observed between the two radiation qualities of 18 and 42 h post-irradiation (*p* < 0.05) (Table 2). Therefore, the fact that no significant difference was observed in residual γ-H2AX foci between ^60^Co γ-rays and neutrons at 18 h could be attributable to the loss of damaged cells, as expected after high-LET radiation exposure. Vral et al. quantified radiation-induced apoptosis by light microscopic analysis and reported no significant difference in apoptosis for PBL exposed to ^60^Co γ-rays and 5.5 MeV neutrons after 24 h, nor at longer culture times of 48–72 h, for doses ranging from 0.05 to 5 Gy^76^. The current study with CD34^+^ cells provides contrasting results, since a significant difference in late apoptosis was observed at 42 h, which indicates that CD34^+^ cells are more prone to undergo apoptosis following high-LET radiation exposure. In a study of Kraft et al*.*, where HSPCs were isolated from peripheral blood of healthy adults, the induction of apoptosis over time was slightly higher for high-LET carbon ions with a maximum of 30–35% for 2 Gy carbon-ions compared to 25% for the same dose of X-rays^26,27^, which is in agreement with a previous study from the same group and the results of the current study with high-LET neutron radiation.

Several studies investigated the apoptosis response of CD34^+^ cells to low-LET radiation. Milyavsky et al. reported approximately 35% of late apoptosis in the progenitor cells (PCs) at 18 h post-irradiation with 3 Gy X-rays, while almost 60% of the pluripotent haematopoietic stem cells (HSCs) were in late apoptosis at the same time point. The majority of the CD34^+^ cells that were used in the current study, are PCs (CD34^+^CD38^+^). Therefore, the late apoptosis results for low-LET ^60^Co γ-rays at 18 h post-irradiation are in close range to the findings of Milyavsky’s group^21^. A The current study shows a twofold higher fraction of late apoptosis in unstimulated CD34^+^ cells at 18 h post-irradiation compared to the 0.5 Gy ^60^Co γ-ray irradiation in the paper of Durdik et al*.* (± 15%)^24^. However, it is important to take into consideration that the background levels (0 Gy) of late apoptosis at 18 h was only ± 10% in the study of Durdik et al*.*, while our values were higher at 18 h (26.24%). However, Durdik et al*.* observed a 2-fold increase late apoptosis (up to 29%), while the current results showed only a slight increase of 8.41%. In general, Durdik et al*.* observed a large increase in late apoptosis with increasing dose from 0.5 to 2 Gy at 42 h, while our results show similar levels of late apoptosis at 42 h irrespective of the ^60^Co γ-rays radiation dose^24^. This comparison with the paper of Durdik et al. indicates that the apoptotic response was somehow quicker in this study and already reached a plateau at 42 h. However, Durdik et al. irradiated the CD34^+^ cells on ice, which might have influenced the DNA damage response at early time points after irradiation. Additional studies with more intermediate time points and different irradiation conditions are needed to clarify the observed differences. A study conducted by Heylmann et al. showed a higher level of basal and radiation-induced apoptosis in freshly unstimulated CD34^+^ cells compared to CD34^+^ cells cultivated in cytokine supplemented medium^72^. The CD34^+^ cells in the current study were not cultured in cytokine supplemented medium for the apoptosis experiments, which could explain the higher background level of apoptosis. The results of Heylmann et al. showed elevated apoptosis rates in T-lymphocytes and CD34^+^ progenitor cells at 24 h following a low radiation dose of 0.125 Gy γ-rays.

This study had several limitations. The CD34^+^ cell populations in this study are a heterogenous mix of primitive HSCs and more lineage-committed PCs, which have differences in radiosensitivity^22^. Future research is needed to elucidate the DNA damage response of the different subsets and how they are linked to specific types of radiation-induced leukaemia after neutron irradiation. In addition, due to the fact that p(66)/Be(40) neutron beam time was not available on demand and has to be prescheduled, we were forced to work with cryopreserved CD34^+^ cells which might have contributed to the variability and the higher level of apoptosis in unirradiated samples, although the error bars are comparable to observations from other groups. As previously mentioned, the CBMN assay allows the detection of DNA damage induced by clastogenic (chromosome breakage) or aneugenic (whole chromosome loss) agents. While it is anticipated that IR induces a predominant clastogenic action, resulting in chromosome breakage, it could be interesting to differentiate between whole chromosome loss (centromere positive MN) and acentric fragments (centromere negative MN) in the CD34^+^ CBMN assay, since only a handful of studies performed the CBMN assay on this cell type and this information is not known yet. Notwithstanding the fact that this is the first study where the DNA damage response of CD34^+^ cells was studied after neutron irradiation, an in vitro study on isolated CD34^+^ cells from UCB is a simplified way to study the underlying mechanisms that are involved in the regulation of HSPC fate and leukaemogenesis. It is difficult to accurately recreate the protective BM niche in vitro as well as the complex interaction of other factors in the human body which might affect the radiosensitivity of HSPCs^77^. Therefore, it might be of key interest to use an animal model to study radiation-induced leukaemia, such as the studies on IR-induced AML CBA/H mice, which are considered to be a reliable model due to their low background incidence and similarities with human AML^78^. Additionally, genomic instability is a common hallmark of radiation-induced malignancies. Particularly in the context of haematopoietic stem cells, the induction of DNA copy number variation (CNV) due to the deletion or duplication of DNA segments, could be one of the underlying mechanisms of radiation-induced leukemogenesis. In order to get a better understanding of the sequence of molecular events that give rise to radiation-induced leukemogenesis, it is important to identify leukemogenic events at chromosomal level, by performing chromosome karyotyping and identifying specific mutations via DNA sequencing. This can be obtained via the tracking of preleukemic cells in vivo as performed by Verbiest et al., who identified a *Sfpi1* point mutation within a subpopulation of isolated preleukemic HSPCs^17^. In addition, the analysis of copy number variation (CNV) via microarray-based genomic sequencing in samples of radiation-induced leukemia patients, might reveal a mutagenic signature which could shed light on the genetic changes that are responsible for leukemogenic event^79^.

In conclusion, this study compared the DNA damage response of CD34^+^ cells exposed to low-LET ^60^Co γ-rays and high-LET p(66)/Be(40) neutrons. A dose dependent increase in apoptosis and cytogenetic damage was observed for both radiation qualities. The high initial number of neutron-induced DNA DSBs and the subsequent higher level of MN formation confirm the higher mutagenic potential of neutrons compared to low-LET radiation, even after a low dose of 0.5 Gy. This study provides valuable information about the deleterious effects on HSPCs of neutron irradiation and its leukaemogenic potential and it opens the scope for future studies to improve our understanding of the molecular mechanisms that are responsible for the observed effects.

## Methods

### Sample collection and isolation of CD34^+^ cells

**Figure 6:**
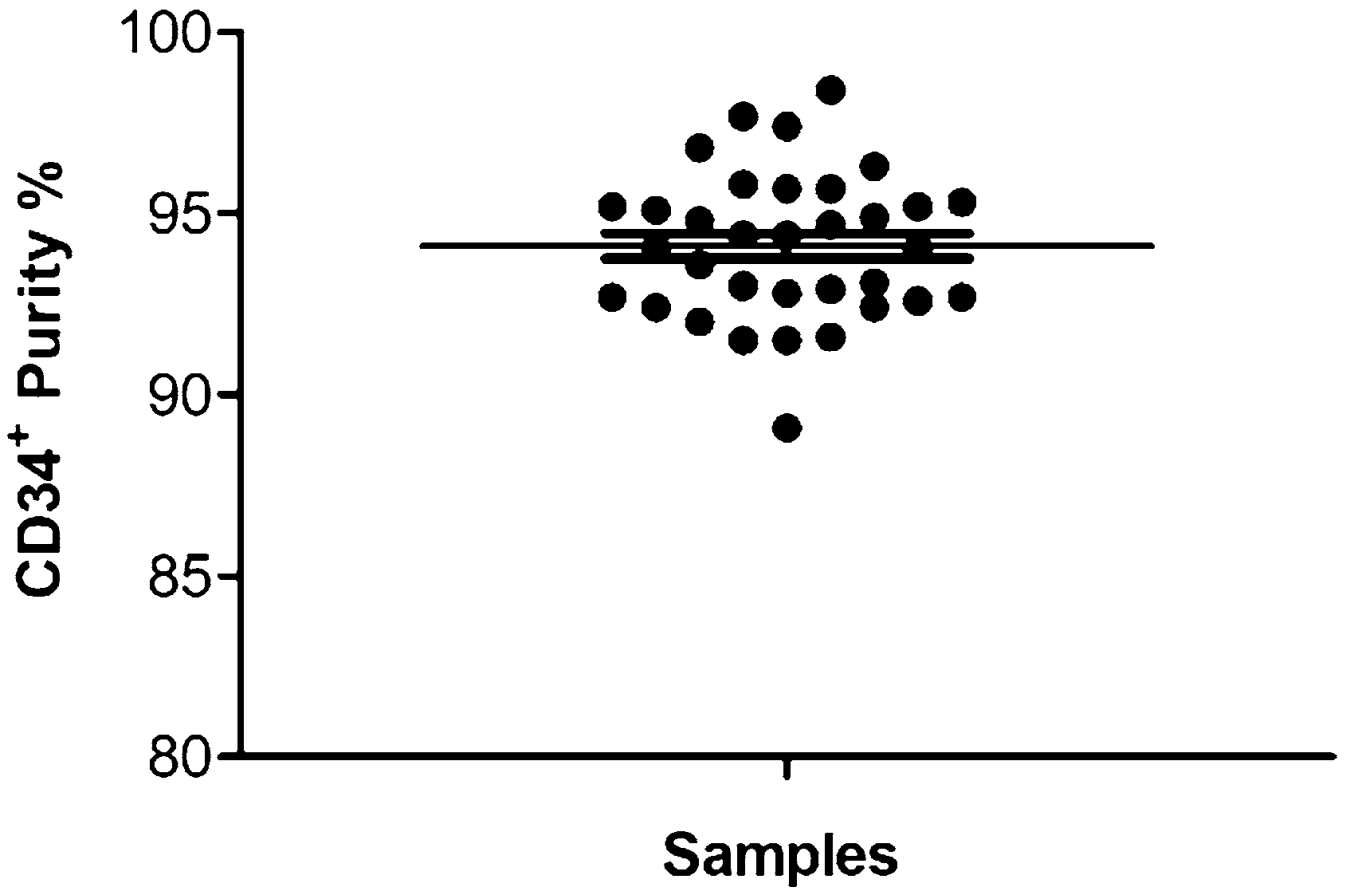
The spread in purity of the CD34^+^ samples as measured with the BD Accuri™ C6 flow cytometer. The average purity of the CD34^+^ cells was 94.11%. The error bar represents the standard error of the mean (SEM) of the different isolated samples (n = 34).

Ethics approval for this study was granted by the Health Research Ethics Committee of the University of Stellenbosch, South Africa (Ethics Reference number: N16/10/134) and all experiments and methods were performed in line with relevant guidelines and regulations. After written informed consent was obtained from each mother, UCB was collected after the scheduled elective Caesarean section. In total, 34 UCB samples (50–90 mL) were collected in bags containing anti-coagulant CPDA-1 (citrate–phosphate–Dextrose–Adenine) (SSEM Mthembu Medical (Pty) Ltd, Cape Town, South Africa) from full-term newborn babies, at either Tygerberg or Karl Bremer Hospital, in Cape Town, South Africa. Samples were transported to iThemba LABS at room temperature, where human CD34^+^ cells were isolated as previously described by Vandevoorde et al.^23^. Briefly, peripheral blood mononuclear cells (PBMCs) were isolated by density gradient centrifugation on Histopaque^®^-1077 (density; 1.077 g/mL; Histopaque^®^-1077, Sigma-Aldrich Co. LLC, St. Louis, Missouri, United States) at 508 RCF for 30 min at room temp and lymphocytes were recoverd from the buffy coat. Next, human HSPCs were purified by using CD34^+^ immunomagnetic beads (Miltenyi Biotec Inc., Bergisch Gladbach, Germany) according to the manufacturer’s recommendations. The isolated CD34^+^ cells were resuspended in 90% fetal bovine serum (FBS) (Gibco, Dun Laoghaire, Dublin, Ireland) and 10% dimethyl sulfoxide (DMSO) (Sigma-Aldrich Co. LLC, St. Louis, Missouri, United States) before − 80 °C storage. A fraction (~ 50 µL containing 50,000 CD34^+^ cells) of each final CD34^+^ sample was used to determine the purity of the isolation using the BD Accuri™ C6 flow cytometer (BD Biosciences). Before the samples were analysed, the CD34^+^ cells were washed with 1% bovine serum albumin (BSA) buffer (Sigma-Aldrich Co. LLC, St. Louis, Missouri, United States), stained with 5 µL of Fluorescein isothiocyanate (FITC) anti-human CD34 monoclonal antibody (Thermofisher Scientific, Massachusetts, United States) and incubated for 30 min at room temperature in the dark. Post incubation, cells were washed with phosphate-buffered saline (PBS) and stained with propidium iodide (PI) (Thermofisher Scientific, Massachusetts, United States) to distinguish dead cells from the viable population. Flow cytometry analysis of 10,000–20,000 CD34^+^ events revealed an average purity of 94.11 ± 0.35% (Fig. 6).

### Irradiation of CD34^+^ cells

#### p(66)/Be(40) neutron irradiations

Approximately 3 h before irradiation, CD34^+^ cells were gradually thawed and resuspended in Iscove's Modified Dulbecco's Medium (IMDM) (Gibco, Dun Laoghaire, Dublin, Ireland), 10% fetal bovine serum (FBS) (Gibco) and 0.5% Penicillin–Streptomycin (Pen-Strep) (Lonza, Walkersville, MD, USA), before being transferred to sterile 2.0 mL cryovials (NEST Biotechnology Co., Ltd., Wuxi, China). Samples were irradiated with a Scanditronix clinical isocentric gantry, where the neutrons are produced by bombarding a thick Beryllium (Be) target with 66 MeV protons generated by the separated sector cyclotron (SSC) at the iThemba LABS Facility (iTL, Cape Town, South Africa). The beam quality was inferred from the neutron energy spectrum with a fluence-weighted average energy of approximately 29.8 MeV for the 29 × 29 cm^2^ field used^80,81^. A hydrogenous filter reduced the contribution of thermal and epithermal neutrons. The source-to-phantom surface distance was 150 cm and irradiations were carried out at a gantry angle of 270°, resulting in a horizontal beam directed onto a water tank containing the CD34^+^ samples at a depth of 5.2 cm in the water. The Perspex wall thickness of the tank was 9.5 mm. Samples were exposed to different doses ranging from 0.05 to 3 Gy at a dose rate of 0.400 Gy/min. Sham-irradiated control samples were maintained in the control room, receiving only ambient radiation. The output factor (1.097 Gy/MU) was measured at the same position as the samples using an Exradin T2 thimble ionisation chamber, with a wall made from A-150 tissue-equivalent plastic with a 0.53 cm^3^ active chamber volume flushed with a propane-based tissue-equivalent gas. The ^60^Co calibration factor used for the cross-calibration of the T2 chamber is traceable to the National Metrology Institute of South Africa (NMISA), while calibrations were performed according to the neutron dosimetry protocol as described in the ICRU Report 45^82^.

#### ^60^Co γ-ray irradiation

In this study, ^60^Co γ-rays were used as a reference radiation quality. The reference dose measurement for the ^60^Co beam were done using the IAEA TRS-398 protocol. The CD34^+^ cell suspensions were irradiated in the 2.0 mL cryogenic vials with ^60^Co γ-rays using a teletherapy unit (Theratron 780). The vials were placed between a 6 mm build-up Perspex plate to ensure dose build-up and a 49.3 mm backscatter plate with a dose rate of 0.468 Gy/min for a 30 × 30 cm^2^ field size. The lateral dimensions of the build-up plate and backscatter block are 299 × 299 mm^2^ and 297.5 × 297.5 mm^2^, respectively. The air gap between the build-up plate and the backscatter block 29.5 mm.The CD34^+^ samples were exposed to radiation doses of 0.05–3.00 Gy depending on the specific assay performed. Sham-irradiated control samples were included for each assay. After irradiations, these samples were incubated at 37 °C, with 5% CO_2_ in 95% humidified atmosphere, until termination time point.

### Cytokinesis-block micronucleus (CBMN) assay

A modified version of the micro-culture CBMN assay that was developed in a previous study was used for these experiments^23^ (modified protocol is courtesy of the Radiobiology research unit at Ghent University). For the CBMN assay, the cells were irradiated with 0.05, 0.5 or 1 Gy of ^60^Co γ-rays or p(66)/Be(40) neutrons. After irradiation, CD34^+^ cells were cultured in a 48-well suspension plate containing 500 µL of complete IMDM supplemented with 10% FBS and 0.5% Pen-Strep and a combination of recombinant haematopoietic cytokines, 100 ng/mL stem cell factor (SCF), 100 ng/mL FLT3 ligand (FL) and 20 ng/mL thrombopoietin (TPO) to stimulate the expansion of the CD34^+^ cells (all cytokines from Miltenyi Biotec Inc., Bergisch Gladbach, Germany). The irradiated cells were incubated as previously described for 70 h. After 23 h, cytochalasin B (CytoB) (0.75 mg/mL) (Sigma-Aldrich Co. LLC, St. Louis, Missouri, United States) was added, which is an inhibitor of microfilament ring assembly that is required for the completion of cytokinesis, which allows to distinguish once-divided cells based on their binucleated (BN) appearance^54^. After 70 h of total culture time, the cells were resuspended gently to reduce cellular clumping and each well was rinsed with PBS. The cell suspension was transferred to Eppendorf tubes, which were then centrifugated at 316 RCF for 8 min at 4 °C (Eppendorf 5810R centrifuge, Hamburg, Germany). Cells were exposed to cold 0.075 M Potassium Chloride (KCl) and an overnight fixation in 3:1:4 (methanol/acetic acid/ringer) solution. The next day, the cells were fixed in 3:1 (methanol/acetic acid) and left at 4 °C overnight. Then, the cells were dropped on isopropanol-cleaned slides and allowed to air dry. Thereafter, slides were stained with acridine orange (100 µL/10 mL, Sigma-Aldrich Co. LLC, St. Louis, Missouri, United States) and MN were manually counted in BN cells using a fluorescent Zeiss Axio Imager A1 microscope (Carl Zeiss AG, Oberkochen, Germany) at 200× magnification. Approximately 500 BN cells were scored per slide (two slides per sample condition). The nuclear division index (NDI) represents the proliferation rate of the cells and was calculated based on the method described by Fenech^54^:

$$ {\text{NDI}} = {{\left( {{\text{M}}_{1} + 2{\text{M}}_{2} + 3{\text{M}}_{3} + 4{\text{M}}_{4} } \right)} \mathord{\left/ {\vphantom {{\left( {{\text{M}}_{1} + 2{\text{M}}_{2} + 3{\text{M}}_{3} + 4{\text{M}}_{4} } \right)} {\text{N}}}} \right. \kern-\nulldelimiterspace} {\text{N}}}, $$

where M_1 _− M_4_ indicate the number of cells with 1–4 nuclei and 500 (N) cells were scored per condition.

### γ-H2AX foci assay

For the γ-H2AX foci assay, unstimulated CD34^+^ cells were suspended in 0.5 mL of complete IMDM. The CD34^+^ cell suspensions were irradiated with 0.5 Gy ^60^Co γ-rays or p(66)/Be(40) neutrons and incubated for 2 or 18 h post-irradiation to allow foci formation and repair. After incubation, the CD34^+^ cells were arrested in ice water for 10 min followed by centrifugation onto coated slides (X-tra adhesive slides, Leica Biosystems, Buffalo Grove, IL, USA) using a Cytospin (Cellspin I, Tharmac^®^ GmbH) in a concentration of approximately 800,000 cells/mL. The two slides were prepared for each exposure condition were fixed in PBS containing 3% paraformaldehyde (PFA) (Sigma-Aldrich Co. LLC, St. Louis, Missouri, United States) for 20 min, followed by overnight incubation in 0.5% PFA in PBS. The immunohistochemistry staining of the resulting slides was performed as previously described by Vandevoorde et al.^23^. Lastly, slides were scored automatically using the MetaCyte software module of the Metafer 4 scanning system (MetaSystems, Altlussheim, Germany) using a 40× objective. Approximately 500–1000 CD34^+^ cells were scored over two slides per condition. The average number of γ-H2AX foci induced by the different radiation qualities was obtained by subtracting the number of γ-H2AX foci derived from the sham-irradiated control samples of each donor from the average γ-H2AX foci number scored in the irradiated samples of the same donor.

### Apoptosis

The isolated CD34^+^ cells were irradiated with 0.5, 1 and 3 Gy of ^60^Co γ-rays or p(66)/Be(40) neutrons and incubated for 18 and 42 h as previously described, to allow the apoptosis process to occur. After incubation, the unstimulated CD34^+^ cell suspension of approximately 100,000 cells/1 mL was centrifuged in a fluorescence-activated cell sorting tube (Corning, New York, United States). The detection of apoptotic cells was achieved using the Annexin V apoptosis detection kit I (Becton Dickinson (BD) Biosciences, New Jersey, United States). Briefly, the concentrated cell suspension was resuspended in 100 µL annexin buffer and stained with anti-Annexin-FITC and PI. After 15 min incubation in the dark, 400 µL annexin buffer (BD Biosciences) was added. Data was acquired on an Accuri™ C6 flow cytometer (BD Biosciences) and approximately 10,000 events were analysed.

### Statistical analysis

The results from the individual experiments were averaged and the corresponding standard error of the mean (SEM) calculated. Statistical analysis was performed using Microsoft Office Excel 2019 (Microsoft Corporation, Washington, DC, USA) and GraphPad Prism Software Version 5.01 for Windows (GraphPad Software, San Diego, CA, USA). FlowJo ™ v10.7 (BD Bioscience) was employed to analyse flow cytometry data. The numbers of experiments (n) are indicated in each figure. The results were obtained over multiple neutron beamtime campaigns and sham-irradiated controls were included at every experiment. Shapiro–Wilk tests assessed normality of the data and Kruskal Wallis test was performed for statistical analysis of the CBMN and apoptosis data. Analysis of variance (ANOVA) was carried out on the NDI and γ-H2AX foci assay data and a significance level of *p* < 0.05 was used in all tests. All statistical tests were 2-sided, and *p*-values < 0.05 (*) were considered statistically significant, *p* < 0.01 (**) highly significant and *p* < 0.001 (***) extremely significant.
